# Reproductive health in Turner’s syndrome: from puberty to pregnancy

**DOI:** 10.3389/fendo.2023.1269009

**Published:** 2023-12-05

**Authors:** Eleonora Porcu, Linda Cipriani, Giuseppe Damiano

**Affiliations:** ^1^ University of Bologna, Bologna, Italy; ^2^ Infertility and IVF Unit, IRCCS Azienda Ospedaliero-Universitaria of Bologna, Bologna, Italy

**Keywords:** Turner syndrome, pubertal development, fertility preservation, mosaicism, oocyte cryopreservation

## Abstract

Turner syndrome (TS) is a genetic pathology that affects about 1/2500 newborn females. Turner’s syndrome is characterized by highly variable genetic anomalies that consist in a partial or complete deletion of the X sexual chromosome; it can be present as a monosomy or as a mosaicism with two o three different cellular lines. 50% of the patients with Turner’s syndrome has a 45 XO karyotype while the remaining cases have karyotypes with mosaicism or X isochromosome or with partial or whole Y chromosome. This pathology is characterized by multiple anomalies that involve physical and cognitive development and in particular endocrine, cardiovascular, reproductive, auditive and visual systems. Integrity of the X chromosome in essential for fertility. In TS is accelerated germ cells apoptosis. About 30% of TS girls have some pubertal development, 10-20% undergo menarche and 2-8% go through spontaneous pregnancy. Women with TS should be informed about the risk of premature menopause and should be referred, if possible, to a specialist evaluation with a doctor expert in assisted reproductive techniques. In adolescents and in adults, Premature Ovarian Insufficiency (POI) can be evaluated clinically and biochemically with the classic combination of amenorrhea and elevated FSH concentrations (hypergonadotropic hypogonadism). However, in postpubertal adolescents and adult women, reproductive hormones may remain within the normal range before POI is clinically evident, despite significant depletion of the ovarian reserve. Today, reproductive medicine offers the opportunity of fertility preservation in women with premature ovarian insufficiency (POI). Two techniques have been suggested such as ovarian cortex cryopreservation and oocytes cryopreservation.

## Introduction

The objective of this manuscript is to review the current knowledge on Turner’s syndrome and reproductive health.

Turner’s syndrome (TS) is a genetic pathology that affects about one of 2,500 newborn girls (1).

Turner’s syndrome is characterized by highly variable genetic anomalies that consist of a partial or complete deletion of the X sexual chromosome; it can be present as a monosomy or as a mosaicism with two or three different cellular lines.

Of the patients with Turner’s syndrome, 50% have a 45 XO karyotype, while the remaining cases have karyotypes with mosaicism or X isochromosome or with partial or whole Y chromosome.

The disease course of premature ovarian insufficiency (POI) differs significantly among the causative genes and the types of mutations. The representative genes whose mutations are significant features in the syndromes and in family history are FOXL2, CLPP, FSHR, and FMR1. Mutations in FOXL2, in the form of BPES type 1, are associated with POI. The fragile X mental retardation gene (FMR1) has been known to be associated with POI. The fully expanded form causes the loss of the RNA-binding FMR1 protein and results in fragile X intellectual disability (2).

This pathology is characterized by multiple anomalies that involve physical and cognitive development, in particular the endocrine, cardiovascular, reproductive, auditory, and visual systems.

Phenotypic characteristics of TS are classic facial appearance, neck webbing, short stature, risk for heart and renal defects, and gonadal dysgenesis with an increased risk of premature ovarian insufficiency (3). The medical care of a girl with TS in a specialized hospital center is complicated due to the complexity of her condition (4).

The integrity of the X chromosome is essential for fertility. In TS, there is accelerated germ cell apoptosis (5, 6). Oocyte apoptosis is accelerated from the early stage of fetal life, and the reduced number of germ cells disturbs primordial follicle development, thereby causing the formation of streak gonads. There are three possible causes of accelerated germ cell loss in 45,X ovaries. First, chromosomal pairing failure due to X chromosomal aneuploidy could induce meiotic arrest. Second, germ cell apoptosis could be caused by impaired coupling between oocytes and granulosa cells. Lastly, ovarian dysfunction in women with TS is partly attributable to the reduced dosage of several genes on the X chromosome (such as BMP15, PGRMC1, and some other genes on the X chromosome) (7). About 30% of TS girls have some pubertal development, 10%–20% undergo menarche, and 2%–8% go through spontaneous pregnancy (8–10).

Women with TS should be informed about the risk of premature menopause and should be referred, if possible, to a specialist evaluation with a doctor expert in assisted reproductive techniques (11).

The combination of amenorrhea and elevated FSH concentrations (hypogonadotropic hypogonadism) can be used to evaluate POI in adolescents and adults (12). However, in postpubertal adolescents and adult women, reproductive hormones may remain within the normal range before POI is clinically evident, despite significant depletion of the ovarian reserve.

Today, reproductive medicine offers the opportunity for fertility preservation in women with POI (13). Two techniques have been suggested, such as ovarian cortex cryopreservation and oocyte cryopreservation (14).

## Diagnosis

The prenatal diagnosis of Turner’s syndrome can be achieved with invasive methods (amniocentesis, chorionic villous sampling), but some ultrasonography features such as increased nuchal translucency, the presence of cystic hygroma, coarctation of the aorta, left-sided cardiac defects, brachycephaly, renal anomalies, polyhydramnios, oligohydramnios, and intrauterine growth retardation should give rise to the suspicion of this syndrome. Abnormal maternal serum screening (alfa-feto protein, beta HCG, inhibin A, and unconjugated estriol) may also suggest the diagnosis of Turner’s syndrome. However, neither ultrasound nor maternal serum screening should be considered diagnostic of Turner’s syndrome, and karyotype confirmation should be mandatory.

The degree of mosaicism prenatally detected is not generally predictive of the severity of the Turner’s syndrome phenotype. Many of the pregnancies with a diagnosis of Turner’s syndrome ended in successful term births.

The American College of Medical Genetics recommends the execution of a standard karyotype at 30 cells in all those born on suspicion of Turner’s syndrome. This analysis allows to identify the cases of mosaicism that involve at least 10% of cells (95% CI). In the case of a high suspicion of hidden mosaicism, other metaphases or studies of fluorescent *in situ* hybridization (FISH) can be performed. Also, probing for the Y chromosome should be performed in all these patients. In cases of virilization in patients with Turner’s syndrome, the search for a possible Y chromosome is necessary, as is the exclusion of a gonads or adrenal gland’s neoplastic pathology. After birth, the diagnosis of Turner’s syndrome should be suspected in all patients who present unexplained retard of pubertal maturation, edema of the hands and feet, retronuchal thickening, left-sided cardiac defect, low posterior hairline, low set ears, small mandible, short stature, elevated Follicle-stimulating hormone (FSH) levels, cubitus valgus, typical facies, multiple pigmented nevi, short fourth metacarpal, high arched palate, and chronic otitis media.

An early diagnosis of this disease allows for the identification of cardiac and auditory defects and pubertal development retardation in order to establish a correct therapy to prevent complications (15).

TS is diagnosed with a standard karyotype counting 15–30 cells. If mosaicism is suspected, it may be necessary to increase the number of cells to 100 cells. In total, 50% of TSs will have a 45,X karyotype, and 40% will have a structural abnormality of the second X chromosome (16).

Most TSs with mosaic karyotypes do not have the classic features of TS. The karyotype study should be performed if short stature, delayed puberty, lymphedema, and aortic co-arctation are present in history (15).

## Pubertal development

Turner’s syndrome is classified among the conditions of POI. The number of germinal cells is normal until 18 weeks of gestation, then the degeneration process begins. From early childhood (2–5 years), increased levels of FSH and LH are measured, and in the adult age, these reach menopausal levels.

TS patients with monosomy X have small, striated gonads and hypogonadotropic hypogonadism, while about 30% of girls with TS with mosaicism have spontaneous puberty, 4% reach menarche, and 1% are fertile (17).

Almost 90% require hormone replacement therapy to ensure progress in puberty, maintain secondary sexual development, and promote bone health (18).

Up to 30% of patients with Turner’s syndrome show signs of pubertal development, and 2%–5% present regular menstrual cycles without therapy; 2% of these patients have a spontaneous pregnancy (19). Recently, some ovarian follicles were observed also in 12–19-year-old patients with monosomy 45 XO. Aso (20) investigated the possibility of predicting spontaneous menarche and regular menstrual cycles in 50 patients with Turner’s syndrome. The patients were divided into three groups: in the first group, patients with spontaneous menarche before 16 years and regular cycles for at least 18 months; in the second group, patients with spontaneous menarche before 16 years but irregular cycles and secondary amenorrhea; and in the third group, patients without spontaneous breast development before 14 years or with primary amenorrhea at 16 years. The authors analyzed the values of FSH and LH in these patients at 12–13 years. The results confirmed that the patients with a karyotype with mosaicism have more frequent regular cycles. The patients with FSH levels lower than 10 mIU/mL at the age of 12 years presented spontaneous menarche and regular menstrual cycles, confirming the significance of FSH levels as a preventive sign of spontaneous menarche and regular cycles, as well as the absence of spontaneous menarche, hypoplastic ovaries, or reduced development of the uterus (21).

The occurrence of spontaneous pubertal development is directly associated with the presence of the second X chromosome in the karyotype; patients with X monosomy have a much lower incidence of spontaneous puberty than patients with mosaicism (21). Purushothaman (22) confirmed that some karyotypes, including monosomy 45 XO, Xq deletions, and 46 XY mosaicism, are associated with poor fertility potential, while other karyotypes, such as 46 XX mosaicism and terminal Xp deletions, are more frequently related to spontaneous menarche.

However, in a recent study by “The US National Institute of Health,” spontaneous pregnancies in women with a 45 XO karyotype, the classic form of Turner’s syndrome, were observed, suggesting that there are different alleles involved in the regulation of fertility, placed in different positions on the X chromosome (**Table 1**) (8).

**Table 1:** Background data and pregnancy outcome in women with IS (8).

Characteristic	Own oocytes	Oocyte donation	All TS
Background data			
**No. of women**	27	30	57
- Spontaneous pregnancy (n)	23		
- IVF (n)	3		
- Insemination (n)	1		
**Age, y, median (range)**	27 (16–42)	37 (24–44)	32 (16–44)
Marital status, n (%)			
- Married/cohabitant	27 (100)	30 (100)	57 (100)
Socioeconomic status, n (%)			
- Student	5 (18)	9 (30)	14 (24)
- Employed	22 (82)	20 (67)	42 (74)
- Unemployed	0 (0)	1 (3)	1 (2)
Chromosomal constitution, n (%)			
- 45,X	1 (4)	16 (53)	17 (30)
- 45,X/46,XX	25 (92)	2 (7)	27 (47)
- Y	1 (4)	3 (10)	4 (7)
- Iso, ring, trisomy X	0 (0)	9 (30)	9 (16)
Pregnancy outcome			
- No. of pregnancies	82	42	124
- Delivery, n (%) (liveborn rate)	36^a^ (44)	31 (74)	67 (54)
- Liveborn children (n)	37	31	68
- Miscarriage, n (%)	37 (45)	11 (26)	48 (39)
- Legal abortion, n (%)	8 (10)	0	8 (7)
- Extrauterine pregnancy, n (%)	1 (1)	0	1 (1)

a One set of twins.

A Swedish study describes 12% of pregnancies in women with Turner’s syndrome (57/482), with a live birth rate of 54% in 124 pregnancies. Approximately 40% (23/57) of pregnancies were spontaneous, 5% (three of 57) were obtained with IVF, 2% (one of 57) were obtained with IUI, and 53% (30/57) with oocyte donation (23).

It is very important to establish the timing of introduction, the type, the dose, and the route of administration of estrogen therapy. Estrogen therapy is initiated around the age of 11–12 years if the gonadotropin level is high and the Anti-Mullerian Hormone (AMH) is low and gradually increased over a 2–3-year period to mimic the physiological increase (24). Therapy begins with half of a 14-mg patch applied weekly or a whole 14- or 25-mg patch for 1 week per month at age 11–12 and escalates every 6–12 months based on response and potential for growth. When estradiol transdermic is not available or compliance is an issue, evidence supports the use of oral micronized estradiol. Delaying estrogen treatment is detrimental to bone development and other aspects of the child’s health. Estrogen doses increase at 6-month intervals and can mimic the normal pubertal period (25).

In girls, growth hormone (GH) therapy is recommended; initiation of GH therapy before low-dose estrogen is important for growth (26).

To minimize the risk of irregular bleeding, endometrial hyperplasia, and endometrial cancer, additional oral progestogen therapy (for 10 days/month or continuously) is required after 2 years of estrogen treatment (27).

## Turner’s syndrome and fertility

Spontaneous pregnancy in TS has an incidence of 2%–7% (28).

Oogenesis and folliculogenesis are compromised in girls with TS; a high rate of abnormal follicles was detected, and follicular atresia was accelerated (29). The presence of follicles depends on karyotype (mosaicism), age, and concentration of FSH and AMH (30) In fact, spontaneous puberty is correlated with high levels of AMH and inhibin B (31).

Spontaneous pregnancies in women with 45,X and mosaic TS are between 2% and 7% (24). The miscarriage rate reported is 31% (32). Spontaneous pregnancies in TS have a high risk of fetal sex chromosome aneuploidy and trisomy 21 and a 3.8% rate of aneuploidy. An increased risk of chromosomal aneuploidy may include premature aging of oocytes (33).

Possible complications of spontaneous pregnancy are aortic dissection due to dilatation of the aorta and cardiac decompensation (34).

## Fertility preservation: evaluation for potential fertility in women with TS

The evaluation for potential fertility in girls with TS consists of:

- Evaluation of karyotype
- Evaluation of ovarian reserve (AMH and antral follicles count).

The karyotype of women with TS was correlated with the size of the ovarian primordial follicle pool and spontaneous puberty; in fact, studies have shown that the chromosomal structure of women with TS has an impact on ovarian function (35).

Ovarian reserve capacity varies among women with TS, so it is important to select patients who can preserve their fertility as early as possible. Predictive parameters are serum AMH level, serum FSH level, karyotype analysis, development of spontaneous puberty, and ultrasound assessment of antral follicle count (AFC). AMH concentrations are higher in patients with spontaneous puberty and mosaic karyotype.

Women with TS should receive an early diagnosis, an assessment of ovarian reserve, and, in cases of residual ovarian function, options for fertility preservation (36).

Fertility preservation can be obtained by four different methods: oocyte donation, oocyte cryopreservation, embryo cryopreservation, and heterologous transplantation of ovarian tissue. The embryo cryopreservation after ovarian stimulation is the only treatment encoded by the ethics committee American Society for Reproductive Medicine (ASRM) to preserve fertility in patients with Turner’s syndrome, but this method cannot be applied in pre-pubertal patients. In these patients, the only possible methods are oocyte and ovarian tissue cryopreservation. The purpose of ovarian tissue cryopreservation is the recovery of primordial follicles in the ovarian cortex before the end of the atretic process in order to culture them *in vitro* (37, 38). Atresia and damage can affect primordial and primary follicles during freezing, and preovulatory antral follicles do not usually survive the procedure of ovarian tissue cryopreservation, whose clinical efficiency should be confirmed (35).

An accurate selection of Turner’s syndrome patients who can be submitted to treatment for fertility preservation is very important. The discussion about the opportunity to do these treatments in patients with karyotypes without mosaicism is still open. Hreinsson (35) suggested that patients aged 12 and 13 should be subjected to ovarian tissue cryopreservation in order to obtain a relevant number of primordial follicles; 90% of the patients with XO karyotype had already had FSH levels above 40 mIU/mL in pre-pubertal age. The ideal age to perform oocyte or ovarian tissue cryopreservation is not yet established, but some authors suggest that the age of 12–14 years is more suitable (39).

Another study (38) suggests that ovarian tissue cryopreservation should be performed before puberty, as at the beginning of puberty, the number of follicles may already have been reduced. Overall, the biopsy is an invasive method that should be reserved only for selected patients; at the beginning, the evaluation of ovarian reserve should be done through noninvasive methods such as plasmatic screening of FSH, AMH, and inhibin B. The extent and rate of decline of the plasmatic level of AMH, the reduction of inhibin B, and the increase of FSH are associated with ovarian failure. In particular, AMH is a predictive marker of ovarian reserve, but the sensibility and specificity of this marker should be assessed by further studies (22). A study by Hagen (30) showed a close association between levels of AMH and ovarian reserve. Turner’s syndrome patients with X monosomy or with the absence of spontaneous pubertal development and premature ovarian failure had already very low levels of AMH before 25 years (AMH < 2–7 pmol/L). On the contrary, patients with mosaicism or with preserved ovarian function had normal levels of AMH.

Another treatment to preserve fertility in these patients is heterologous transplantation of ovarian tissue. Mhatre and Mhatre (40) described the first case of transplantation of ovarian tissue from the mother to a 15-year-old daughter with Turner’s syndrome. The menstrual cycles have returned for 12 months, but the effects of immunosuppressive therapy and ovarian function in the following years should be assessed through additional studies.

Lau et al. (41) recently described the application of oocyte cryopreservation in patients with Turner’s syndrome by analyzing the reproductive state of 28 patients with Turner’s syndrome; 46% of the patients had a partial or complete deletion of the X chromosome, 32% had mosaicism, and 21% had X isochromosome or X ring chromosome.

The age of diagnosis was variable; 21% of cases were diagnosed at prenatal or neonatal age, 21% between 6 months and 10 years, and 57% between 11 and 20 years. Six patients (21%) had spontaneous pubertal development: five of these had mosaicism, whereas one with monosomy X had reached the stage of development Tanner 2. Thus, 14% (all with mosaicism karyotype) had a spontaneous menarche. The ultrasound exam showed in seven of 21 patients regular uterine and ovarian morphology; only one of these patients had a karyotype with X monosomy. The levels of FSH were examined in 21 patients and were associated with karyotype. Approximately 10 of 11 patients (91%) with karyotype 45 XO had levels of FSH greater than 40 IU/mL, whereas two of four patients with X isochromosome or X ring chromosome had levels of FSH lower than 40 IU/mL. The mean plasma concentration of FSH in the group with mosaicism karyotype resulted significantly lower than in the group with karyotype with monosomy X or X ring chromosome (25.9 IU/L ± 10.4 IU/L vs. 80.7 IU/L ± 8.0 IU/L and 53.9 IU/L ± 19.2 IU/L, *p* < 0.05). The increase in FSH levels directly correlated with increasing age in the patients with karyotype 45 XO: the increase in FSH levels above 40 IU/L appeared at the age of about 16 years. Based on the criteria listed above, four patients (14%) were suitable for fertility preservation. The features of these patients and their reproductive history are shown in **Tables 2**, **3**.

**Table 2:** Characteristics of the four patients with Turner’s syndrome who are potentially eligible for fertility preservation (41).

Patient No.	Age at diagnosis (years)	Karyotype (%)	FSH (IU/L)	Age at FSH measurement (years)	Ultrasonographic findings	Spontaneous puberty (Tanner stage)	Age of spontaneous menarche
1	16	45,X (74/46),XX (247), XXX (24)	NA	NA	1 ovary and uterus seen	5	13
2	14	45.X (98)/47,XXX (2)	7.7	14	1 ovary and uterus seen	4	13
3	10	45.X (6¥46), XХ (94)	7.1	10	Both ovaries and uterus seen	5	11
4	9	45,X (98)47,XXX (2)	6.6	15	Both ovaries and uterus seen	5	14

NA, non available.

**Table 3:** Current reproductive status and fertility outcome of four potential fertility preservation candidates (41).

Patient No.	Current age (years)	Current reproductive status and fertility outcome
1	31	Spontaneous pregnancy at the age of 24 years; hormone replacement therapy for 6 years
2	31	Hormone replacement therapy for 15 years
3	23	Normal menses ranging 30–40 days
4	18	Normal menses; ovarian stimulation and oocyte cryopreservation at the age of 16

One of the four selected patients (patient 4) has undergone ovarian stimulation with a GRH agonist and recombinant FSH at 450 IU for 5 days and then 600 IU for the other 5 days. Two oocytes at the stage of metaphase II were recovered at the oocyte retrieval and were vitrified (41). Moreover, El-Shawarby (39) treated a patient with mosaic Turner’s syndrome who was 22 years old and obtained eight oocytes that were vitrified for future use. These results confirm previous observations of Abir (38): the preservation of fertility should be suggested to patients with mosaicism or X isochromosome. However, the discussion about the patients with X monosomy is still open.

Similarly, further evidence is needed to identify the appropriate age for oocyte cryopreservation.

## Oocyte cryopreservation

This technique is no longer considered experimental (42); therefore, oocyte preservation is now one of the best choices to preserve fertility in cancer patients (43–47). In recent years, this technique has been used for various medical, social, ethical, and legal indications.

Oocyte cryopreservation has several safety reports, and more than 3,000 live births have been achieved with no evidence of an increase in infantile abnormalities (48).

With regard to efficiency, fertilization of cryopreserved oocytes, pregnancy, and delivery outcomes are like those of fresh *in vitro* fertilization (IVF) (44, 48).

Oocyte cryopreservation is a safe and effective technique to protect fertility, even in women with mosaic TS (49). This technique has been used successfully in a small number of women with TS, and the number of mature oocytes retrieved in a single cycle is very small. Therefore, the procedure must often be repeated to finally obtain more than 10 mature oocytes. The patients’ oocytes have been found morphologically and chromosomally normal (50).

The first live birth using cryopreservation of own oocytes was recently published (51).

In total, 31 patients with TS received fertility counseling at the Infertility and IVF Unit, IRCCS Azienda Ospedaliero-Universitaria di Bologna, Italy, between 2000 and 2022. Karyotype, spontaneous menarche, and menstrual rhythm were evaluated during the counseling. The ovarian reserve was determined by measuring serum AMH, FSH, and ultrasound AFC. Seven patients (mean age 23.2 years ± 3.9 years) underwent ultrasound-guided oocyte retrieval after controlled ovarian stimulation. The mean value of AMH was 0.6 ng/mL ± 0.4 ng/mL, the mean value of FSH was 12.1 IU/mL ± 3.6 IU/mL, and the mean antral follicles count was 3.4 ± 2.1. Gonadotropins associated with gonadotropin-releasing hormone (GnRH) agonist (leuprolide acetate) or antagonist (cetrorelix acetate) were used to perform ovarian stimulation. Ovulation was triggered with recombinant hCG. Ovarian stimulation was monitored with seriated estradiol blood tests and pelvic ultrasounds. When follicles reached a diameter of 16–17 mm and the estradiol serum levels were considered appropriate, ovulation was triggered with hCG 36 h prior to transvaginal ultrasound-guided oocyte retrieval. The mean number of oocytes retrieved was 2.7 ± 1.3, and the mean number of mature (MII) oocytes stored was 2.2 ± 0.7 per patient.

The selection of patients suitable for cryopreservation can be done through the evaluation of predictive factors for the ovarian follicles’ presence, as hypothesized by Borgström (52). In total, 57 patients with Turner’s syndrome, aged between 8 and 19.8 years, underwent laparoscopic ovarian biopsy. In 15 patients (26%), some follicles in the analyzed ovarian tissue were recovered (86% with mosaicism, 10.7% with X monosomy), whereas eight of 13 patients (62%) with spontaneous menarche and 11 of 19 (58%) with spontaneous pubertal development had some follicles. The higher percentage of follicles was in female patients aged between 12 and 16 years. The patients with some ovarian follicles had more often normal values of FSH and low levels of AMH.

Therefore, spontaneous pubertal development, mosaicism, and normal hormone levels are significantly associated with a higher probability of having ovarian follicles; consequently, they can be used as screening factors to identify patients suitable for ovarian biopsy. Recently, Huang (53) proposed to combine ovarian tissue cryopreservation and oocyte cryopreservation to preserve the fertility in Turner’s syndrome. He described a case of a 16-year-old patient with Turner’s syndrome (karyotype 45,X(20%)/46 XX(80%)) submitted to oocyte retrieval of 11 immature oocytes from the ovarian cortex; eight of these oocytes were vitrified after *in vitro* maturation (maturation rate: 73%) (53).

Oocyte cryopreservation is also a feasible technique for fertility preservation in selected postpubertal female children at risk for premature ovarian failure due to accelerated follicle loss in Turner’s syndrome. However, further studies would be needed to test the results of oocyte cryopreservation in young girls (54).

## Ovarian tissue cryopreservation

This procedure can be performed at any age without the need for spontaneous puberty or gonadotropin stimulation (55). The TS procedure is still experimental; no live births have been reported (56).

## Oocyte donation

Oocyte donation is another method to preserve fertility in Turner’s syndrome.

Oocyte donation is most frequently used in TS (33). The clinical pregnancy rate of oocyte donation is 30%–46%, with a low percentage of miscarriages (23). Compared to conventional IVF, pregnancies achieved with donated oocytes are associated with a higher incidence of gestational hypertension and preeclampsia (57).

The study by Press (58) showed a similar pregnancy rate for cycles between patients with Turner’s syndrome and patients with idiopathic premature ovarian insufficiency in cycles of oocyte donation. Khastgir (59) confirmed the efficacy of this method and obtained a 41.2% pregnancy rate for cycles and a 17% implantation rate for embryos in patients with Turner’s syndrome through oocyte donation. Moreover, the study demonstrated a positive predictive value of the endometrial thickness on the day of embryo transfer: there is a higher pregnancy rate for the cycle when the endometrium is thicker than 6.5 mm.

## Pregnancy in Turner’s syndrome

The application of methods to preserve fertility in Turner’s syndrome should be associated with suitable counseling about the risks of pregnancy in these patients. Maternal and fetal complications are increased in TS pregnancies. The possible risks are diabetes, placental insufficiency, metabolic disease, preeclampsia, and intrauterine growth restriction (60). The risk of fetal loss and congenital malformations is increased both in spontaneous pregnancies and in pregnancies obtained through oocyte donation. In a review of Tarani (61), including 160 spontaneous pregnancies in patients with Turner’s syndrome, the incidence of fetal loss, chromosomal abnormalities, congenital malformations, and perinatal death was respectively 29%, 20%, and 7%.

Birkeback (62) analyzed the reproductive state of 412 women with Turner’s syndrome by consulting the dates of the Danish register; 33 of these women (only one with 45 XO karyotypes, 27 with mosaicism, and five with structural anomalies of the second X chromosome) have given birth to 64 children. The karyotype of 25 children out of 64 was analyzed, and in six cases, a chromosomal anomaly was diagnosed (24%). Other studies show a higher incidence of trisomy 21 (4% vs. 0.4% in the general population) and Turner’s syndrome (15% vs. 0.5% in the general population) in patients with Turner’s syndrome (63–65). The increased incidence of chromosomal anomalies in these fetuses could be one of the possible causes of the increased abortion rate; other favoring factors are uterine malformations, reduced uterine development, reduced uterine vascular perfusion, reduced endometrial receptivity, and the presence of autoantibodies that correlate with the higher incidence of autoimmune pathologies in the Turner’s syndrome patients. Further studies (59) documented a high incidence of miscarriages in patients with Tuner’s syndrome who underwent cycles of oocyte donation. This finding confirms that the major incidence of uterine anomalies in these patients represents an important cause of miscarriage. The age of the recipients in cycles of oocyte donation did not seem to influence the success rates.

Bakalov (66) evaluated the association between uterine development in Turner’s syndrome and hormone therapy. The normal uterine development observed in Turner’s syndrome patients with spontaneous pubertal development would exude the existence of an intrinsic uterine defect that previous studies had suggested (67). The uterine development in Turner’s syndrome seems rather to be related to the administration of hormone replacement therapy (HRT), as was demonstrated in 86 women with Turner’s syndrome, aged 18–45, who underwent an ultrasound examination of the uterus. About 1/4 (24.4%) of the women had a normal uterine development, while most (44.2%) had a small uterus and 1/3 (31.4%) had an immature uterus. The patients who took HRT had a significantly larger uterus than the patients who used oral contraception or without therapy. Duration and typology of HRT influenced uterine development; treatments including estradiol showed the highest efficacy.

The age of first exposure to estrogens, the stature and weight of the patients, and the previous intake of growth hormone do not seem to be related to uterine measures, but previous studies recommended initiation of estrogen therapy at the age of 12–15 years.

Karyotype was not associated with uterine dimensions, unlike suggested in previous studies (66, 68) that showed a correlation between normal uterine development and mosaicism (**Table 4**).

**Table 4:** Variables influencing uterine volume (66).

Continuous indenendent variables			
	*R* ^2^	*F* value	*p*-value
Age	0.24	5.08	0.027
Height	0.008	0.68	0.411
Weight	0.017	1.46	0.230
Body surface area	0.016	1.40	0.240
Age at estrogen exposure	0.022	1.85	0.177
Years of estrogen exposure	0.161	15.94	0.0001
Nominal independent variables			
	Level	Volume (mL; mean ± SD)	*p*-value
History of GH use	Yes (*n* = 26)	18.3 ± 12.0	0.066
	No (*n* = 60)	22.8 ± 12.3	
Current HRT	Yes (*n* = 69)	23.0 ± 12.6	0.0019
	No (*n* = 17)	15.0 ± 8.6	
Spontaneous menarche	Yes (*n* = 13)	30.0 ± 16.9	0.014
	No (*n* = 73)	19.9 ± 10.7	
Type of estrogen	E2 (*n* = 10)	28.0 ± 10.0	E2 vs. none, *p* = 0.0015
	CE (*n* = 28)	25.0 ± 14.6	E2 vs. OC, *p* = 0.045
	OC (*n* = 27)	19.7 ± 11.0	CE vs. none, *p* = 0.0032
	None (*n* = 20)	15.4 ± 8.6	

E2, estradiol; CE, conjugated estrogens; OC, oral contraceptives.

The available dates for the pregnancies of Turner’s syndrome patients are still limited. A retrospective study by Bodri (69) analyzed the outcomes of the pregnancies of 21 patients with Turner’s syndrome who underwent 30 cycles of oocyte donation between 2001 and 2004. Among the 17 pregnancies obtained, 12 were clinic, with a high rate of biochemical miscarriage (29% vs. 12.9% in the general population). The implantation and pregnancy rate were respectively 22% (15 of 68) and 30% (nine of 30). The premature birth rate was 50%, and intrauterine growth retardation was found in 55.5% of fetuses. Hypertension was diagnosed in five of eight pregnancies, and there were three cases of preeclampsia. The increased incidence of hypertensive disease in this group of patients, observed also in previous studies (69, 70), is not related to increased maternal age nor the high incidence of multiple pregnancies as in the general population who undergo oocyte donation.

All the patients underwent cesarean sections because of preeclampsia (two cases) and fetopelvic disproportion (the remaining). Fetopelvic disproportion has been described as the main indication for cesarean section in different studies (8, 71).

These data suggest the opportunity for careful monitoring of the pregnancies obtained in Turner’s syndrome and the necessity to reduce the multiple-pregnancy rate that is associated with an increased rate of hypertensive disease. Mortality for cardiovascular complications, in particular aortic dissection, is threefold increased in women with TS compared to the general population. The risk of death from aortic dissection in TS is two of 1,000 (27).

The risk of maternal death in a pregnancy obtained through oocyte donation is about 2%; seven cases of dissection of the aorta during the pregnancy were reported in the literature. The hyperdynamic and hypervolemic vascular state associated with the pregnancy seems to increase the risk of dissection of the aorta. Moreover, gravidical hyperestrogenism can change the structural integrity of the aorta and make it more susceptible to damage. The risk of aortic dissection is increased in the early weeks of the pregnancy and in the third trimester.

Boissonas (72) reported a case of a patient with Turner’s syndrome (45 X0–46 XY karyotype) who had a pregnancy from oocyte donation. The patient did a cardiac screening before the pregnancy that resulted in normal but had dissection of the aorta at 38 weeks of gestation after the diagnosis of the bicuspid aortic valve at 16 weeks of gestation. ASRM recommends a cardiac screening in all patients with Turner’s syndrome who want a pregnancy (73). The screening should include echocardiography, ECG, and magnetic resonance. The detection of a severe cardiac anomaly should represent a contraindication to assisted reproduction in this group of patients. In patients with Turner’s syndrome who undergo ART, a single embryo transfer should be performed in order to avoid the hemodynamic overload associated with multiple pregnancies (15). Recently, the French College of Obstetricians and Gynecologists (FOG) brought together a committee that included the French Societies of Obstetrics and Gynecology, Cardiology, Cardiac Surgery and Vascular Surgery, Anesthesia, Endocrinology, the Study Group on Oocyte Donation, and the Biochemical Agency, with the aim of establishing guidelines about the management of the patients with Turner’s syndrome before and during the pregnancy.

The recommendations include a list of the exams required before the pregnancy, information for the patients, indications about monitoring the pregnancy and kind of delivery, and postnatal follow-up (74).

It is essential to perform a multidisciplinary evaluation before pregnancy with a team including maternal–fetal medicine specialists and cardiologists to evaluate the possible risks (7, 75, 76).

## Conclusions

In TS, the reproductive consequences are primary amenorrhea and premature ovarian insufficiency. Estrogen replacement therapy should be started around the age of 12 to reduce the morbidities related to hormone deficiency. After menarche, the option of oocyte cryopreservation should be offered to patients with mosaic Turner’s syndrome. Pregnancy is a high risk in TS; management of pregnancy with a multidisciplinary specialist team should be implemented in order to reduce complications for mothers and infants.

## Author contributions

EP: Conceptualization, Data curation, Formal Analysis, Investigation, Methodology, Project administration, Resources, Software, Supervision, Validation, Visualization, Writing – original draft, Writing – review & editing. LC: Conceptualization, Data curation, Formal Analysis, Investigation, Methodology, Writing – original draft, Writing – review & editing. GD: Conceptualization, Data curation, Formal Analysis, Investigation, Methodology, Writing – original draft, Writing – review & editing.
